# Comparison of different methods to obtain and store liver biopsies for molecular and histological research

**DOI:** 10.1186/1476-5926-8-3

**Published:** 2009-07-08

**Authors:** Gaby Hoffmann, Jooske Ijzer, Bas Brinkhof, Baukje A Schotanus, Ted SGAM van den Ingh, Louis C Penning, Jan Rothuizen

**Affiliations:** 1 Department of Clinical Sciences of Companion Animals, Faculty of Veterinary Medicine, University Utrecht, Yalelaan 104, 3584 CM Utrecht, the Netherlands; 2 Department of Pathobiology, Faculty of Veterinary Medicine, University Utrecht, Yalelaan 104, 3584 CM Utrecht, the Netherlands; 3 TCCI Consultancy BV, Utrecht, the Netherlands

## Abstract

**Background:**

To minimize the necessary number of biopsies for molecular and histological research we evaluated different sampling techniques, fixation methods, and storage procedures for canine liver tissue. For addressing the aim, three biopsy techniques (wedge biopsy, Menghini, True-cut), four storage methods for retrieval of RNA (snap freezing, RNAlater, Boonfix, RLT-buffer), two RNA isolation procedures (Trizol and RNAeasy), and three different fixation protocols for histological studies (10% buffered formalin, RNAlater, Boonfix) were compared. Histological evaluation was based on hematoxylin-eosin (HE) and reticulin (fibrogenesis) staining, and rubeanic acid and rhodanine stains for copper. Immunohistochemical evaluation was performed for cytokeratin-7 (K-7), multidrug resistance binding protein-2 (MRP-2) and Hepar-1.

**Results:**

RNA quality was best guaranteed by the combination of a Menghini biopsy with NaCl, followed by RNAlater preservation and RNAeasy mini kit extraction. These results were confirmed by quantitative RT-PCR testing. Reliable histological assessment for copper proved only possible in formalin fixed liver tissue. Short formalin fixation (1–4 hrs) improved immunohistochemical reactivity and preservation of good morphology in small liver biopsies.

**Conclusion:**

At least two biopsies (RNAlater and formalin) are needed. Since human and canine liver diseases are highly comparable, it is conceivable that the protocols described here can be easily translated into the human biomedical field.

## Background

Expression profiling can be used for disease classification, predictions of clinical outcome or the molecular dissection of affected pathways in hereditary or acquired diseases. Animal models for human diseases facilitate cause-effect studies under controlled conditions and allow comparison with untreated or healthy individuals. Especially the latter can be an ethical or logistic problem in human medicine. More than 300 genetic human disorders are described in dogs . Many of these diseases occur in one or just a few of around 400 dog breeds. Single gene diseases are easy to characterize in inbred dog populations, and research of complex diseases profits from the fact that dogs share the human environment. In addition to similarities between dogs and humans with respect to physiology, pathobiology, and treatment response, research of breed-related canine behaviour and phenotypic diversity is promising. Therefore dogs were advocated as a model animal in translational research [1]. Molecular genetic tools available for such comparable research between dogs and humans include the in-depth sequencing of the complete dog genome [2,3], a single-nucleotide polymorphism (SNP) data base, containing 2.5 million SNPs [4], and easy access to genetic information of several generations of dogs. In addition, the high degree of inbreeding, which founded the present dog breeds the last few hundreds years, further facilitates the investigations in inheritable gene defects [5-7]. Dog specific micro-arrays are available to perform functional genomic studies. This kind of high-throughput gene expression profiling requires the use of high quality mRNA. Likewise is the quality of mRNA of major impact on the reliability of the results in quantitative RT-PCR (Q-PCR). So far the emphasis in canine molecular biology was put on the use of internal controls for proper Q-PCR measurements and subsequent data analysis [8-10]. However, little information is available that compares different methods of retrieval, isolation and storage of canine tissues for molecular research purposes. Especially liver, but also heart and jejunum, are difficult tissues for retrieval of high quality mRNA [11].

Liver biopsies, taken for medical and research purposes, are processed for histopathology including immunohistochemistry and RNA and protein isolation. Since these diverse intentions require different fixation and storage methods, clinicians and researchers are often faced with a multitude of different vials, and fluids in order to retain biopsies. In addition, the applications of specific fixation protocols can be necessary, which might require additional training, time and sophisticated laboratory equipment. Such complexity of tissue handling can challenge the operating personnel, and therefore introduce mistakes, especially in the setup of a multi-centre study, where sampling procedures should be as straightforward as possible. Moreover, in small lesions or advanced diseases, the possibility for retrieval of several biopsies can be limited.

One study described the influence of the size of the biopsy needle in rat liver biopsies on the RNA quality in a subsequent micro-array expression study [12]. The aim of our study was to assess different sampling techniques (with the optimal needle size as described above), fixation methods, and storage procedures for canine liver tissue. Our objective was to optimize the use of a single liver biopsy, in order to minimize the number of necessary biopsies per patient, by evaluation of different methods for RNA isolation and fixation available in our laboratory. Three biopsy techniques (wedge biopsy, Menghini, and True-cut), four storage methods for retrieval of RNA (snap freezing, RNAlater, Boonfix, RLT-buffer), two RNA isolation procedures (Trizol and RNAeasy), and three different fixation protocols for histological studies (10% formalin, RNAlater, Boonfix) were compared. Histological evaluation was based on hematoxylin-eosin (HE) and reticulin (fibrogenesis) staining, and rubeanic acid and rhodanine stains for copper. Immunohistochemical evaluation was performed for three different proteins at different (sub)cellular locations keratin-7 (K-7), multidrug resistance binding protein-2 (MRP-2) and Hepar-1.

## Results

### RNA isolation: RNAeasy mini kit versus Trizol

The A260/A280 ratios of all samples in this study were between 1.98 and 2.13. The RNAeasy mini kit isolation was compared to the Trizol mediated isolation protocol in RNAlater fixed Menghini biopsies. RNA-quality of RNA isolated with the RNAeasy mini kit was consistently superior (1 to 1.5 RIN-values higher) to RNA isolated with the Trizol method (Table 1). Results from assessment of RNA quality prompted us to restrict further comparisons of different RNA fixation protocols to RNA isolated with the RNAeasy mini kit.

**Table 1:** RIN-values after RNA isolation with RNAeasy mini kit or Trizol method (data of three independent representative isolations).

**RNAeasy**	**Trizol**
8.1	7.3
8.8	7.4
8.2	6.7

Biopsy was taken with True-cut technique, RNA was stored in RNAlater. Independent samples were split and divided over the two isolation procedures.

### Tissue fixation for RNA isolation

RNA quality was compared between four methods of biopsy fixation: snap-freezing, Boonfix, B-RLT medium, and RNAlater. Table 2 depicts a comparison for RNA quality after RNA isolation with the RNAeasy mini kit. Three independent results per fixation protocol were measured. Snap-freezing, B-RLT, and RNAlater revealed RIN-values consistently within the range required for micro-array (range 7.9 to 9.3). A slight tendency for higher RIN-values for blind biopsies compared to True-cut biopsies. Since the RNA isolated from liver tissue fixed in Boonfix had RIN-values often below 8 (range 7.1–8.1), we excluded Boonfix from further molecular analysis.

**Table 2:** RIN-values after RNA isolation with RNAeasy kit after different fixation protocols.

	minus 70°C	Boonfix	B-RLT	RNAlater
True cut (dry)	7.9	7.0	8.7	9.2
	8.7	7.3	8.6	8.5
	8.4	7.2	8.2	8.6
Blind biopsy (NaCl)	8.1	8.1	9.1	9.1
	9.1	7.4	9.3	9.2
	9.0	7.1	9.0	8.5

### Biopsy technique

RIN-values of True-cut derived RNA were slightly lower then biopsies retrieved by the Menghini technique. The difference in RIN-values was around 1 (Table 2).

The effect of the solution used during the Menghini technique on RNA quality was evaluated in RNAlater preserved/RNAeasy mini kit isolated material. The use of Menghini water was compared to Menghini NaCl. Biopsies for this comparison were retrieved from surplus tissue obtained from one research dog, allowing both measurements of RNA quality and quantity. The RNA yield of Menghini NaCl was more than 5 fold higher than Menghini water. The RNA quality however was comparable (RIN-values above 8). Comparison of RNA quality obtained from biopsies of patients revealed superior quality of Menghini NaCl biopsies compared to Menghini water sampling (RIN-values up to 8.8 compared to around RIN-values of 6 resp.).

### Fixation time

For liver tissue kept in RNAlater additional comparisons were made to reveal a possible influence of the time interval from biopsy retrieval to carry over to the preservative. Time lags of 15, 20, 25, and 30 minutes between biopsy retrieval with the Menghini NaCl method and complete enclosing of the biopsy with RNAlater did not affect RNA quality or quantity. In addition freezing of liver biopsies kept in RNAlater at minus 20°C up to 18 months did not affect RNA quality or quantity.

### Gene expression

**Figure 1:**
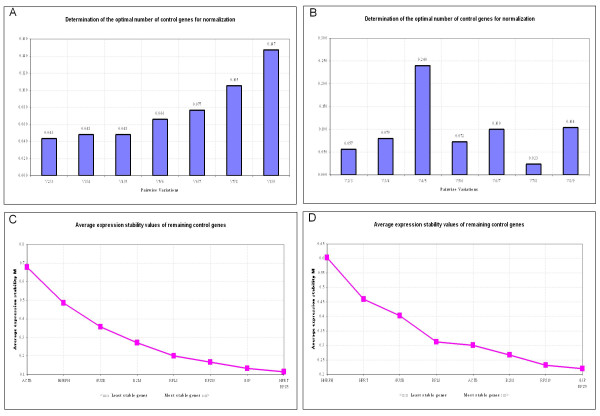
**Determination of the optimal number of reference genes for normalization**. The GeNorm program calculates average expression stability (M) and the expression stability value by the calculation of the pair wise variation. For example V5/V6 indicates the variation in normalization factor with 5 versus 6 reference genes. A and C: Menghini NaCl. B and D: Menghini water.

The optimal number of reference genes for normalization for both Menghini biopsy techniques was determined using the GeNorm program . The analysis was based on the following reference genes: beta-Actin, B2M, GAPDH, GUSB, HNRPH, HPRT, RPL8, RPS19, and RPS5, as previously described [8]. This analysis was slightly in favor for Menghini NaCl above Menghini water, since the pairwise variation (V) was lower and more stable over a wide range of reference genes (Figure 1A, B). In both situations GAPDH, RPS5 and RPS19 are amongst the most stably expressed reference genes (Figure 1C, D).

### Histology

Three different fixation protocols (included 10% neutral buffered formalin, Boonfix, and RNAlater) designed for histological studies were compared.

Histological evaluation of 24 hrs formalin fixed wedge biopsies revealed normal liver histology in healthy dogs. One dog revealed chronic passive congestion with centrolobular hepatocellular atrophy and a severe non-specific reactive hepatitis. Two dogs showed normal hepatic architecture with moderate hepatocellular yellow-brown pigment granulation (copper) in zone III and II and in dispersed Kupffer cells. Hepatitis was not present. Positive copper control dog had severe chronic active hepatitis with a copper score of 3+.

**Figure 2:**
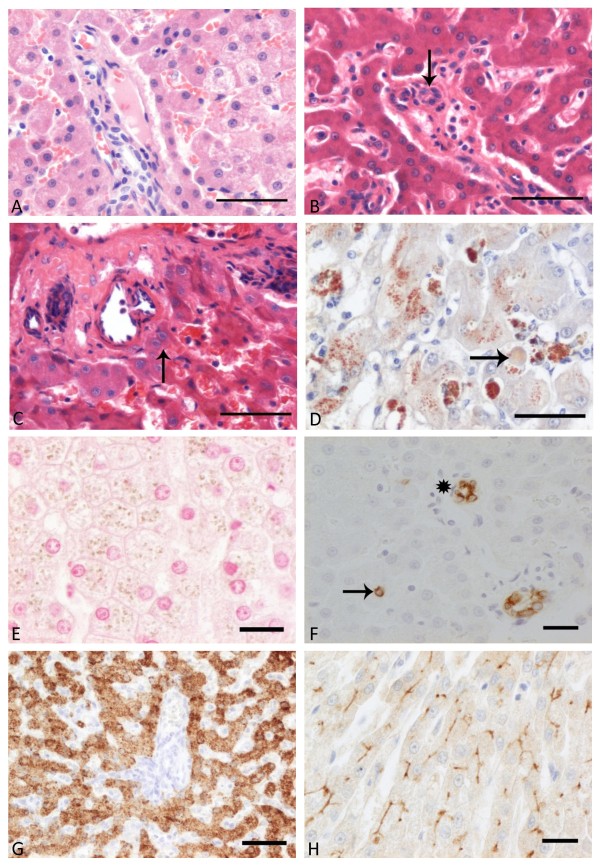
**Liver histology**. A) Normal liver, dog #1, portal area and periportal parenchyma. The tissue architecture is well preserved, with good contrast and sufficient cellular morphology reflected in distinct cellular and nuclear membranes, and sufficient cytoplasmic details. Needle biopsy, 1 h formalin fixation, HE staining, bar 50 μm. B) Normal liver, dog #5, portal area with bile duct (arrow) and periportal parenchyma. The tissue is well conserved, and there is mild cellular pronounciation and slightly enhanced eosinophilic appearance of all cells save erythrocytes. Needle biopsy, 8 hrs Boonfix fixation at room temperature, HE staining, bar 50 μm. C) Normal liver, dog #5, portal area and periportal parenchyma. Insufficient conservation of tissue architecture in the central part of the biopsy, to the right hand side of the arrow, with ill defined cellular borders, strong eosinophilia and shrinkage of hepatocytes, pycnotic nuclei and artificially widened sinusoids. Needle biopsy, 8 hrs RNAlater fixation at room temperature, HE staining, bar 50 μm. D) Copper related chronic active hepatitis, dog #9, parenchyma, control tissue. Many, black staining copper granules appear in the cytoplasm of hepatocytes and Kupffer cells. Wedge biopsy, 24 hrs formalin fixation, rhodanine acid stain, bar 50 μm. E) Liver with copper storage, dog #6, parenchyma. Intracytoplasmic copper granules stain yellow-brown, therefore no reliable differentiation between copper and lipofuscin granules can be made. Needle biopsy, 8 hrs Boonfix fixation, rubeanic acid stain, bar 50 μm. F) Normal liver, dog #2, portal area and periportal parenchyma. Cholangiocytes in the portal tract (asterisk) display a strong signal (brown) in the cytoplasm with negligable aspecific background staining. Also, the parenchyma contains one small, isolated positive periportal cell (arrow), interpreted as a progenitor cell. Needle biopsy, 1 h formalin fixation, K-7 immunohistochemistry, bar 20 μm. G) Normal liver, dog #5, portal area and periportal parenchyma. All hepatocytes feature strong cytoplasmic reactivity, all other cells are negative. Needle biopsy, 1 h formalin fixation, Hepar1 immunostaining, bar 50 μm. H) Normal liver, dog #8, parenchyma, control tissue. Strong signal (brown) is elicited along the canalicular membranes of all hepatocytes, insignificant background staining. Wedge biopsy, 24 hrs formalin fixation, MRP-2 immunostaining, bar 20 μm.

HE staining was consistent in all formalin fixed slides regardless of duration of the fixation, which varied from 1 hr to 5 days (data not shown). There was well preserved tissue architecture, cellular morphology and detail (Figure 2A). Delay of fixation by 30 min storage in NaCl 0.9% did not sort any negative effect. In Boonfix preservative, independent of fixation time, the tissue was well conserved with mild cellular pronunciation, and a mildly enhanced eosinophilic cellular appearance of all cells save erythrocytes which manifested as non-reacting shadows (Figure 2B). Pigmentation in hepatocytes and Kupffer cells was comparable to that seen after formalin fixation. Insufficient tissue preservation occurred centrally in the RNAlater fixed biopsies. Here, cellular borders were ill-defined accompanied by strong eosinophilia and shrinkage of hepatocytes with condensed nuclear chromatin (pycnotic nuclei) and widened sinusoids also containing cells with pycnotic nuclei (Figure 2C). In the well preserved periphery of the biopsy, pigment granules (ceroid/lipofuscin) in hepatocytes and Kupffer cells appeared similar as in formalin fixation. Storage in minus 20°C did not alter the appearance for Boonfix or RNAlater treated tissue sections. Reticulin staining accentuated the interstitial reticulin fibres strongly and uniformly in all formalin fixed slides, irrespective of the duration of fixation or delay of fixation by storage for 30 min in 0.9% NaCl. Boonfix treated slides stained similarly. In RNAlater, histomorphologic changes in the central core were as described above. In the well preserved periphery of the sections reticulin fibers stained strongly.

### Copper staining

Rhodanine stained wedge liver biopsies of copper related hepatitis displayed intensely stained red copper granules in the hepatocellular cytoplasm and Kupffer cells. However, in formalin fixed and RNAlater treated Menghini biopsies copper granules stained yellow-brown to faintly red, so no reliable differentiation with lipofuscin pigment was achievable. Boonfix treated biopsies exhibited only yellowish copper granules.

In standard rubeanic acid staining many positive black copper granules were present in the hepatocellular cytoplasm and in Kupffer cells of the positive formalin fixed control wedge biopsy (Figure 2D). Copper granules in the biopsies stained positive (black) in formalin fixation, but appeared yellowish in both Boonfix (Figure 2E) and RNAlater treated sections, thus differentiation with lipofuscin granules was not possible. Enhancement of the rubeanic acid stain for copper by previous washing in formalin did not change the appearance and staining of these granules; previous treatment with HCl rendered all tested sections negative, including the positive control.

### K-7

Formalin fixed sections showed specific brown, granular cytoplasmic staining of cholangiocytes and periportal progenitor cells with negligable background staining, comparable to previous canine studies [13,14] (Figure 2F). Strongest intensity appeared centrally in the 24 hrs fixed wedge biopsy, with a prominent decrease of signal to the periphery of the section. Menghini needle biopsies showed the strongest and most consistent signal up to 3 hrs of formalin fixation. With longer fixation, the signal decreased, but remained present up to 5 days of formalin fixation. Delay of fixation by immersion for 30 min. in 0.9% NaCl diminished the signal significantly. Boonfix treated slides varied within slides from negative to positive independent of fixation time and also showed increased background staining when compared to formalin fixed tissue. After 8 hrs storage in minus 20°C no reactivity was left. A strong signal was present in the well preserved areas of RNAlater conserved specimens, with extension of background reactivity to all hepatocytes. Storage in minus 20°C did not change reactivity.

### Hepar1

Independent from fixation time or the 30 min delay of fixation, formalin fixed slides stained for Hepar1 rendered strong to very strong granular cytoplasmic staining in all hepatocytes and occasionally some background reactivity on blood plasma (Figure 2G). However, 8 hrs formalin fixed biopsies displayed an irregularly dispersed signal throughout the slide, while the biopsy fixed over 5 days reacted as the biopsies fixed up to 4 hrs. The control tissue revealed strongly increased reactivity in individual periportal hepatocytes, which was less obvious in the Menghini biopsies. Both Boonfix and RNAlater fixed specimens, also after minus 20°C storage, showed a strong signal in the periphery of the biopsy, but reacted very poorly in the centre.

### MRP-2

In 24 hrs formalin fixation, the positive control wedge biopsy exhibited a strong brown signal along the canalicular membranes of all hepatocytes for MRP-2, with negligible background staining (Figure 2H). Increase in fixation time up to 5 days significantly decreased reactivity in a wedge biopsy. Menghini biopsies fixed from 1 h up to 5 days generally proved negative, with some faint signal at 4 hrs. All Boonfix treated specimens were negative. RNAlater preserved specimens had a moderate to strong signal at the periphery of the biopsy, unless stored at minus 20°C after which no signal was present.

## Discussion

In search for an easy-to-use method to acquire, fix and store canine liver biopsies, we used the stability of 18S and 28S rRNA as markers for totalRNA and mRNA stability. Histological evaluation was based on HE, reticulin, rhodanine and rubeanic acid stains and three different immunohistochemical stains.

RNA quality was best guaranteed by the combination of a Menghini biopsy with NaCl, followed by RNAlater preservation and RNAeasy mini kit extraction. Under optimal biopsy conditions (as was the case for the surplus dog used to compare Menghini NaCl and Menghini water in one single dog), no differences in RIN-values between the two techniques were observed. Whether this reflects the fact that exactly the same liver was used, or whether time delay between the biopsy and the actual RNAlater storage, as usually occurs under clinical situations, causes this difference remains unknown. In favor for the first explanation accounts that in the clinical setting the difference was consistent over a large number of biopsies. The evaluation of the optimal number of reference genes needed to obtain reliable data strengthened the observation that the combination of a Menghini NaCl biopsy followed by RNAlater preservation and an RNAeasy mini kit extraction yields optimal RNA quality from canine liver biopsies. The size of the biopsy needle used in this study was based on a previous study on rat liver biopsy techniques, and turned out to be an optimal balance between quantity and quality of the biopsy and the health risks for the animal [12]. This approach of RNA retrieval proved to be a rapid and feasible method for storage for further molecular analysis, and is in agreement with the findings of others for yeast, human renal and uterine myometrial tissues [15-17]. The quality of the obtained RNA in our approach was feasible for micro-array analysis, which requires the highest possible RNA quality, preferential a RIN value above 8.0. Unfortunately our results show that optimal RNA stabilization was only achieved with media that were unsuitable for histology or immunohistochemistry. Histology of RNA later treated biopsies, evaluated in HE and reticulin staining turned out to be of insufficient quality; furthermore, for the antibodies tested either the background staining was too high or central staining appeared very poor.

The best fixative for (immuno)histochemistry proved to be 10% neutral buffered formalin. Boonfix fixation gave good morphology and results in routine HE and reticulin staining, but was suboptimal for the tested immunohistochemical staining methods. RNAlater fixation yielded poor morphology in routine histology and in immunohistochemistry. Most likely, these shortages in morphological evaluation of RNAlater treated specimens were related to insufficient tissue fixation. Boonfix treated specimens generally evoked less intense reactivity immunohistochemically, but as all tested methods were optimized for use in formalin fixed (24 hrs) wedge biopsy specimens, they might perform better in a study where the protocols are tailor-made to the fixative. Storage in minus 20°C for Boonfix and RNAlater, as required for molecular purposes, significantly worsened tissue morphology.

In our experience staining artefacts more frequently occur in small formalin fixed paraffin embedded biopsies. We hypothesized that in the relatively small biopsies overfixation could easily occur. Therefore an effect of the duration of formalin fixation was assessed with subsequent immunohistochemical evaluation of antibodies to proteins at three different (sub)-cellular locations in addition to routine histological staining methods. Differences of the immunohistochemical reactivity for all three antibodies were found between wedge biopsies and the smaller Menghini tissue samples in this study. The observation was most pronounced in MRP-2 stained slides where only a very weak signal was evoked in the smaller biopsies. In addition prolonged fixation in formalin caused a signal reduction for K-7, but did not affect routine HE and reticulin staining. The difference is most likely due to changes in epitopes required for immunohistochemistry, but less for routine HE and reticulin staining. Indications for possible overfixation by formalin were present in K-7 and possibly in MRP2 staining. Signal reduction in K-7 stained biopsies was associated with increased fixation time and was also present in the periphery of wedge biopsies (24 hrs and 5 days fixation). In both situations, prolonged exposure to formalin could explain epitope masking due to protein cross linking of the tissues antigens. Consequently, this antigen masking could result in decreased antigen-antibody reactivity. Occurrence and intensity of this effect will vary per antibody as not all epitopes will be affected similarly [18]. Immunohistochemical reactivity was optimal after formalin fixation and replacement of the formalin by ethanol 70% within 1 – 4 hrs.

Formalin fixation proved necessary for assessment of copper accumulation in liver tissue. Routine rubeanic acid staining was sufficient in a wedge biopsy (24 hrs) as well as in a Menghini biopsy (8 hrs). Reliable rhodanine staining was limited to a wedge biopsy only. RNAlater or Boonfix treated slides did not produce a sufficient signal in any of the investigated copper stains. Interestingly, previous exposure to HCl damp in rubeanic acid staining, as was suggested to enhance copper staining [18], completely inhibited the signal in all slides and therefore proved to be ineffective.

## Conclusion

Summarized, in the search to decrease the number of biopsies needed for molecular and (immuno)histochemical analysis, it turned out that at least two biopsies (10% neutral buffered formalin and RNAlater) are needed. Since both biopsies can be dispersed in relatively non-toxic liquid preservatives, this combination can easily provide researchers with material for high throughput expression analysis. Moreover it nicely resembles the sample preparation protocols that are commonly used in clinics today. Since biopsies fixed in either RNAlater or formalin remain stable at room temperature, transport is easy from the clinical situation to the research facility for further processing as well as prolonged storage. Results of our study showed that a reduction of the formalin fixation time to 1 to 4 hrs will generally reduce formalin induced reduced staining and staining artifacts. Therefore, any extension of the formalin fixation period should be discouraged when immunohistochemistry is considered.

In view of the large similarities between human and canine liver diseases [19], it is conceivable that the protocols described here can be easily translated into the human biomedical field. Consequently, unique and rare human liver biopsies can be obtained, stored and subsequently handled without loss of information.

## Methods

### Animals

All procedures were approved by the responsible ethical committees according to Dutch legislation.

For this study, liver tissue was obtained from seven dogs. In addition two archival specimens were used as positive controls for staining during histologic examinations. Surplus animals from orthopedic research revealed, histologically confirmed, healthy livers. These dogs were euthanized immediately prior to extirpation of the liver, using an overdose of pentobarbital via the cephalic vein.

### Liver biopsies

Liver biopsies were taken according to the Menghini technique described by Rothuizen [20] and by use of a 16-gauge biopsy needle using an automatic biopsy device (Pro-Mag Ultra Automatic Biopsy Instrument, PBN Medicals, Stenløse, Denmark). Liver biopsies retrieved by use of the Menghini technique were kept in physiologic saline solution (0.9% NaCl in sterile water, group Menghini NaCl) or sterile water (group Menghini water) until transfer into according preservatives. Liver biopsies retrieved with the True-cut gun were kept at room air until transfer into the different storage media.

After fixed time periods the material was further processed with either one of the following four methods: snap freezing and subsequent storage at minus 70°C, transfer into a sterile 1.5 ml vial containing 1 ml of RNAlater (Applied Biosystems, Nieuwerkerk a/d lJssel, the Netherlands), Boonfix (Finetec, Tokyo, Japan) or B-RLT (QIAGEN, Venlo, the Netherlands). Biopsies in these vials were kept at 4°C for 2 hrs, and later transferred to minus 20°C and minus 70°C freezing for long-term storage (2 weeks to 18 months). Additional biopsies retrieved exclusively for histologic examinations were retrieved by the Menghini-NaCl method, and immediately deposited at room temperature (RT) per three in 6 ml containers filled with 10% neutral buffered formalin. Wedge biopsies (1 × 1 × 1 cm) were put in a larger container, containing at least 10 cm^3 ^of formalin.

### Isolation of RNA, reversed transcriptase and quantitative RT-PCR

RNA isolations with the RNAeasy kit (QIAGEN) or Trizol reagent (Invitrogen, Leek, the Netherlands) were performed according to the manufactures instructions. RNA yields were quantified spectrophotometrically using the Nanodrop ND-1000 (Isogen Life Science, IJsselstein, the Netherlands) device and set to a 0.1 μg/μl concentration. One microgram of each total RNA sample was used to synthesize cDNA with an MMLV-derived reverse transcriptase according to manufacturer's protocol (iScript cDNA synthesis kit, Bio-rad, Veenendaal, the Netherlands). Details were described previously [19].

RNA quality was measured in two independent ways: By means of the A260/A280 ratio, which estimates the amount of protein contamination, and by means of the Agilent 2100 Bioanalyzer (Agilent Technologies, Amstelveen, the Netherlands), which displays RNA Integrity Number (RIN-values) indicating the percentage of intact 18S and 28S rRNA.

A SYBR Green based quantitative PCR was performed on a Bio-Rad My-IQ detection system as described previously [8].

### Histology

After the specified fixation times (range 1 hr to 5 days), formalin was replaced by 70% ethanol until further processing. Other tissues were immersed in RNAlater (8 hrs) and Boonfix (2, 4, 8 hrs). In addition, also a biopsy fixed in RNAlater or Boonfix was kept in a minus 20°C freezer prior to further processing. After the different fixation procedures and replacement of preservatives by ethanol all tissue samples of one individual animal were simultaneously dehydrated and paraffin embedded. Paraffin blocks were stored at 4°C until use.

Routine histology performed on 3 μm sections included HE (all animals save two controls), and the reticulin staining according to Gordon and Sweet (5 dogs). Primary histological evaluation was based on the 24 hrs formalin fixed wedge biopsies. Two cases with known hepatic copper storage were also subjected to routine rhodanine and rubeanic acid stains for copper accumulation. Moreover, two enhancement methods of rubeanic acid staining [18] were performed by 1): washing the slides 5 min. in 10% neutral buffered formalin previous to rubeanic acid staining, or 2): after de-waxing, slides were placed face downwards over a beaker of HCl 37% for 15 min., followed by 15 min. wash in ethanol 90% and routine rubeanic acid staining. The copper scoring system was described previously [21]. Single immunohistochemical staining for K-7, Hepar1, and MRP2 was performed as previously described [13,14].

## Abbreviations

B2M: beta-2 microglobulin; GAPDH: glyceraldehyde-3-phosphate dehydrogenase; GUSB: β-Glucuronidase; HE: hematoxilin-Eosin; hnRNPH: Heterogeneous nuclear ribonucleoprotein H; HPRT: Hypoxanthine phosphoribosyltransferase; K-7: cytokeratin-7; MRP-2: multi drug resistance protein-2; Q-PCR: quantitative real-time PCR; RPL8: ribosomal protein L8; RPS19: ribosomal protein S19; RPS5: ribosomal protein S5; SNP: single nucleotide polymorphism.

## Competing interests

The authors declare that they have no competing interests.

## Authors' contributions

GH performed the biopsies and wrote the first draft of this manuscript. JIJ performed the IHC and co-wrote the first draft of this manuscript. BB and BAS did the molecular analysis. TSGAMvdI evaluated the histology. LCP and JR designed the experimental set-up and co-wrote the final version. All authors have read and approved this manuscript.
